# Association between 1,5-Anhydroglucitol and Acute C Peptide Response to Arginine among Patients with Type 2 Diabetes

**DOI:** 10.1155/2020/4243053

**Published:** 2020-07-21

**Authors:** Yun Shen, Yiming Si, Jingyi Lu, Xiaojing Ma, Lei Zhang, Yifei Mo, Wei Lu, Wei Zhu, Yuqian Bao, Gang Hu, Jian Zhou

**Affiliations:** ^1^Department of Endocrinology and Metabolism, Shanghai Clinical Center for Diabetes, Shanghai Diabetes Institute, Shanghai Key Laboratory of Diabetes Mellitus, Shanghai Jiao Tong University Affiliated Sixth People's Hospital, Shanghai, China 200233; ^2^Pennington Biomedical Research Center, Baton Rouge, Louisiana, USA 70806

## Abstract

**Objective:**

The aim of this study was to explore the association of 1,5-anhydroglucitol with acute C peptide response (ACPR) to arginine among patients with type 2 diabetes.

**Methods:**

Patients with type 2 diabetes were enrolled from the Department of Endocrinology and Metabolism, Shanghai Sixth People's Hospital. ACPR was assessed using arginine stimulation test. Decreased *β*-cell function was defined as ACPR < 2.1. Multivariable logistic regression models were used to demonstrate the association between 1,5-anhydroglucitol and decreased *β*-cell function.

**Results:**

Finally, 623 patients with type 2 diabetes were enrolled into the analysis. Multivariable-adjusted odds ratios for decreased *β*-cell function across quartiles of 1,5-anhydroglucitol were 1.00, 0.47 (95% confidence interval (CI) 0.23-0.99), 0.41 (95% CI 0.20-0.84), and 0.27 (95% CI 0.13-0.57) (*P*_trend_ = 0.042), respectively. When 1,5-anhydroglucitol was considered as a continuous variable after logarithm, the corresponding odds ratio was 0.40 (95% CI 0.23-0.71).

**Conclusions:**

We demonstrated a dose-response linear association between 1,5-anhydroglucitol and ACPR. 1,5-Anhydroglucitol was likely to be associated with *β*-cell function. Further analysis with large sample size and prospective study design is warranted to validate our findings.

## 1. Introduction

The prevalence of type 2 diabetes was reported to be 10.9% in China [1], imposing a heavy burden on public health and healthcare costs. Clinical studies have suggested that the pathogenesis of type 2 diabetes is usually considered as insulin resistance in which pancreatic *β*-cells increase their mass and insulin secretion as the initial step [2, 3]. The subsequent step is decreased insulin secretion due to *β*-cell dysfunction and potential *β*-cell death, leading to hyperglycemia [4]. A recently reported study demonstrated that *β*-cell metabolism-secretion coupling could be decreased by glucotoxicity via a novel mechanism [5]. Glucose levels, the pulsatile secretion of insulin, insulin secretion stimulated by glucose or other substances such as amino acids, and some substances secreted by *β*-cells such as proinsulin, have been used to assess *β*-cell dysfunction [6, 7]. We have previously applied the arginine stimulation test (AST) in our clinical center during a long period of time [8]. AST has been usually used to evaluate the acute-phase insulin secretion function of *β*-cells, and in this test, the insulin secretory response is stimulated by arginine rather than glucose. The AST is easy to perform and well tolerated since it requires only a few minutes and does not result in hyperglycemia. Previously, we have demonstrated that acute C peptide response (ACPR) evaluated by the arginine stimulation test may be superior to other commonly used *β*-cell function parameters to reflect glycemic fluctuation in insulin-treated patients with type 2 diabetes [9].

Any kind of stimulation test is invasive. It is interesting to find a glycemic indicator that can be closely associated with *β*-cell function in the clinical practice. 1,5-Anhydroglucitol [10], as a new marker for postprandial glycemic control within one to two weeks, is less influenced by diets or physical activities than point glycemic markers. 1,5-Anhydroglucitol is usually used as an effective supplementary marker to hemoglobin A1c. In addition to the role as a measure of hyperglycemic exposure, 1,5-anhydroglucitol has been shown to be related to glycemic excursions. We previously reported that 1, 5 − anhydroglucitol × glycated hemoglobin A1c/100 can be used as a potential marker for *β*-cell function among patients with type 2 diabetes [11]. Based on this finding, we further hypothesize that 1,5-anhydroglucitol can also well correlate with the acute insulin response assessed by AST. Therefore, the aim of this study was to explore the association of 1,5-anhydroglucitol with acute insulin response to arginine among patients with type 2 diabetes.

## 2. Methods

### 2.1. Study Subjects

We consecutively enrolled 623 successive patients (437 men, 186 women) who were admitted to the Department of Endocrinology and Metabolism, Shanghai Jiao Tong University Affiliated Sixth People's Hospital, from January 2015 to the end of September 2019 and were diagnosed as type 2 diabetes according to the 2010 American Diabetes Association standards, which has been described previously. Inclusion criteria included age ≥18 years and presence of type 2 diabetes. Additionally, patients who used insulin secretagogues, dipeptidyl peptidase 4-inhibitors, and sodium-glucose cotransporter-2 inhibitors or had severe hepatic dysfunction, renal dysfunction, malignant tumor, pregnancy, or mental disorders were excluded (see supplementary figure). This study was approved by the Ethics Committee of Shanghai Jiao Tong University Affiliated Sixth People's Hospital, Shanghai, China. All participants signed the informed consent.

### 2.2. Clinical Measurements

A standardized questionnaire on general information of examination date, birth date, sex, and medication history was conducted through a face-to-face interview. At admission, trained nurses measured height, body weight, and blood pressure using a standard protocol.

### 2.3. AST

ACPR (equally to acute insulin response) [12] was determined using intravenous AST under fasting conditions, when the fasting plasma glucose (FPG) concentration had been stable and was less than 12.6 mmol/L. After a baseline blood sample was collected, a 10% (wt/vol.) solution of arginine hydrochloride (5 g) (Shanghai Xinyi Jinzhu Pharmaceutical Co., Ltd., Shanghai, China) was injected intravenously for 30-45 s. At the end of the injection, blood samples were obtained at 2, 4, and 6 min. The ACPR to arginine was calculated as the mean of the 2, 4, and 6 min C-peptide values minus the baseline values. ACPR < 2.1 was defined as decreased *β*-cell function (acute phase) [13].

### 2.4. Mixed-Meal Tolerance Test and Biomarker Measures

During the hospitalization, all patients were instructed to adhere to a standard diet. This diet was designed to ensure a total daily caloric intake of 25 kcal/kg, with 55% of caloric coming from carbohydrates, 17% from proteins, and 28% from fats. A venous blood specimen was drawn in the next morning after hospital admission with 10-12-hour fasting. Biochemical parameters including fasting C-peptide (FCP), plasma glucose, glycated hemoglobin A_1c_ (HbA_1c_), total cholesterol (TC), triglyceride (TG), high-density lipoprotein cholesterol (HDL-c), and low-density lipoprotein cholesterol (LDL-c) were determined as described previously. Postload blood samples were collected to assess 2-hour plasma glucose (2hPG) after the participants ate a mixed meal. Serum creatinine was used to calculate estimated glomerular filtration rate (eGFR) by the Modification of Diet in Renal Disease (MDRD) [14].

### 2.5. Statistical Analysis

Statistical analyses were performed using SPSS 24.0 software package (SPSS Inc., Chicago, IL, USA) and SAS for Windows, version 9.3 (SAS Institute, Inc., Cary, North Carolina, USA). Categorical variables were expressed as a percentage (%). Normally distributed variables were presented as the mean ± standard deviation, and nonnormally distributed data were expressed as the median with the interquartile range. The Jonckheere-Terpstra test was for nonnormally distributed data to perform the trend analyses. With a cutoff threshold value of ACPR < 2.1 to separate normal from decreased *β*-cell function, the multivariable logistic regression analysis was conducted to identify the independent association of 1,5-anhydroglucitol with decreased *β*-cell function. The analyses were first carried out adjusting for age and then further for sex, diabetes duration, BMI, SBP, TG, LDL-c, HDL-c, and eGFR. Similarly, multivariable linear regression analysis was used to determine the independent association between 1,5-anhydroglucitol and ACPR. Restricted cubic spline nested in logistic regression models was performed to test whether there were dose response or nonlinear associations of 1,5-anhydroglucitol as continuous variables with odds of decreased *β*-cell function. A two-tailed *P* value of < 0.05 was considered statistically significant.

## 3. Results

**Figure 1:**
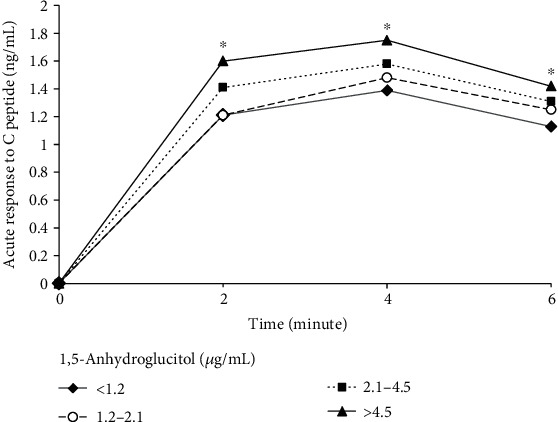
Acute response to C peptide across different quartiles of 1,5-anhydroglucitol. ^∗^*P* for trend <0.05 across quartiles of 1,5-anhydroglucitol at different time points.

Six hundred twenty-three eligible patients were studied in the final analysis. Overall, 1,5-anhydroglucitol levels were higher in women than those in men (*P* < 0.05). In addition, when patients in this study were divided by age of 45 years old, 1,5-anhydroglucitol levels were also found higher among patients over 45 years old than patients younger than 45 years old (*P* < 0.05). The baseline characteristics of the study subjects are further listed in Table 1 by quartiles of 1,5-anhydroglucitol. Age, duration of type 2 diabetes, HDL-c, and ACPR increased across quartiles of 1,5-anhydroglucitol (all *P* for trend <0.001). The trend of other glycemic markers such as HbA_1c_, GA, FPG, and 2hPG was consistent across quartiles of 1,5-anhydroglucitol. Patients with lower 1,5-anhydroglucitol tended to be insulin-treated and were less likely to use oral glucose-lowering drugs.  Figure 1 showed ACPR across different quartiles of 1,5-anhydroglucitol at different time point. For each time point (2, 4, and 6 minutes), ACPR also showed an increasing tendency with the increase of 1,5-anhydroglucitol levels (all *P* for trend <0.05).

Multivariable adjusted odds ratios for decreased *β*-cell function across quartiles of 1,5-anhydroglucitol were 1.00, 0.47 (95% confidence interval (CI) 0.23-0.99), 0.41 (95% CI 0.20-0.84), and 0.27 (95% CI 0.13-0.57) (*P*_trend_ = 0.042), respectively (Table 2). When 1,5-anhydroglucitol was considered as a continuous variable after logarithm, the multivariable adjusted odds ratios for decreased *β*-cell function was 0.40 (95% CI 0.23-0.71).

To further validate the association between 1,5-anhydroglucitol and ACPR, multivariate linear regression analysis was performed. 1,5-Anhydroglucitol was shown to be one of the independent impacting factors of ACPR (*β* = 0.421 [0.278 to 0.563], *P* < 0.001) (Table 3).

**Figure 2:**
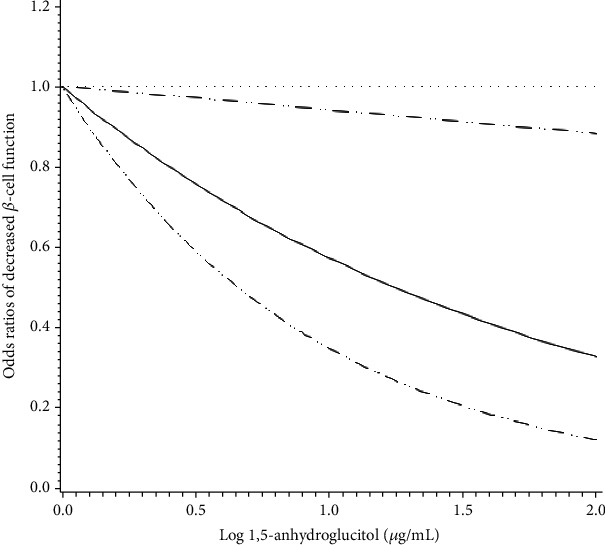
Odds ratios of decreased *β*-cell function by 1,5-anhydroglucitol as continuous variables. Adjustments were made for sex, diabetes duration, BMI, SBP, TG, LDL-c, HDL-c, eGFR, and glucose-lowering treatments other than insulin. 1,5-Anhydroglucitol was transformed by logarithm before analysis.

To well illustrate the dose-response association between 1,5-anhydroglucitol and decreased *β*-cell function, a cubic restricted spline curve was drawn as Figure 2. As a result, a graded negative association between 1,5-anhydroglucitol and the risk of decreased *β*-cell function defined by ACPR < 2.1 was clearly shown.

## 4. Discussion

In this cross-sectional analysis among patients with type 2 diabetes, we found a graded negative association between 1,5-anhydroglucitol and the risk of acute phase *β*-cell function. Patients with type 2 diabetes who had higher 1,5-anhydroglucitol also had lower odds of decreased *β*-cell function assessed by arginine stimulation test. On the other hand, 1,5-anhydroglucitol, as a biomarker for glycemic control within one week, can also partly reflect the acute phase *β*-cell function.

*β*-Cell dysfunction and insulin resistance comprise the main pathogenesis of type 2 diabetes. The death of *β*-cell and the subsequent decreasing *β*-cell function are the key factors of the development of type 2 diabetes [15]. In normal individuals, insulin secretion shows a characteristic biphasic pattern that consists of a transient first phase followed by a sustained second phase [16]. The reduced acute insulin response is the major reason for postprandial hyperglycemia [17]. Intravenous glucose tolerance test and oral glucose tolerance test are commonly used in the clinical practice for assessment of *β*-cell function. However, under circumstance of hyperglycemia, the response of *β*-cell function to insulin is reduced, and it is thus inaccurate to assess the *β*-cell function by glucose challenge [18]. In addition to glucose, some amino acids and fatty acids can also regulate the secretion of insulin. AST, initially found by Ward et al. in 1984 [19], is proved effective and safe in reflecting the acute phase *β*-cell function. The principle of glucose lowering therapies is to preserve the residual *β*-cell function. Therefore, when one patient was firstly diagnosed with type 2 diabetes, it was important to assess his or her *β*-cell function to help decision-making on drugs. It is also of great interest to look for a glycemic biomarker that can simultaneously indicate the degree of *β*-cell function. 1,5-Anhydroglucitol is a newly identified marker showing tight association with glycemic control within one week, which is also superior to HbA_1c_ and glycated albumin on postprandial glycemic levels [20]. In our previous study, we found that 1,5-anhydroglucitol was not only correlated with insulin sensitivity and secretion but also closely associated with early-phase insulin secretion in Chinese patients with type 2 diabetes [21]. However, in the present study, all markers were calculated after glucose challenge, and the acute insulin response was not involved. We thus hypothesized the positive association between 1,5-anhydroglucitol and acute insulin response, which was definitely validated in this study.

Although a significant association of BMI with ACPR was observed, we believe that insulin sensitivity may be an important mediator of this phenomenon. As overweight/obesity is linked to insulin resistance, the beta-cells need to secrete more insulin/C-peptide to maintain the same glucose levels as in lean subjects. However, we could only expect this relationship in an observational study. Cohort studies have already revealed that obesity may be associated with the rate of insulin secretion capacity decline [22]. The causal relationship between the alleviation of obesity and the recovery of *β*-cell function could also be proved based on the evidence from bariatric surgery [23]. Furthermore, the assessment of insulin resistance by HOMA2-IR remains validation in Chinese population.

The underlying mechanism linking 1,5-anhydroglucitol and *β*-cell function is unclear. One recent metabolomic study suggested 1,5-anhydroglucitol as a biomarker that was closely associated with decreasing functional *β*-cell mass before the onset of diabetes [24]. Another Korean study has also shown that low serum levels of 1,5-anhydroglucitol in individuals with prediabetes were associated with lower insulinogenic index but not with higher HOMA-IR [25]. 1,5-Anhydroglucitol is a naturally occurring polyol discovered from foods and cannot be metabolized in vivo [26]. The primary pathway for 1,5-anhydroglucitol disposal is via urinary excretion, while hyperglycemia can promote urinary excretion resulting in the lowering of plasma levels of 1,5-anhydroglucitol [27]. Accordingly, it has been suggested that 1,5-anhydroglucitol is sensitive in reflecting postprandial glycemic excursions [28]. In a study in terms of the role of Prohibitin family in glucose metabolism, the decreased glucose clearance in *β*-Phb2^−/−^ (*β*-cell-specific Prohibitin-2 knockout mice) secondary to *β*-cell failure may contribute to reduced renal reabsorption of 1,5-anhydroglucitol and lowering of its plasma levels [29]. In parallel, the decrease in hepatic 1,5-anhydroglucitol concentrations observed in *β*-Phb2^−/−^ mice could be the consequence of lower glycogen-derived biosynthesis, reducing its efflux normally occurring across the cell membrane [30, 31]. These mechanisms may contribute to the lowering in plasma 1,5-anhydroglucitol levels, reflecting the progressive decline of the functional *β*-cell mass. While ACPR assessed by arginine stimulation test itself cannot be used to assess the *β*-cell mass, studies linking 1,5-anhydroglucitol and *β*-cell mass could be a potential direction for explanation of our cross-sectional findings. Moreover, it has been reported that 1,5-anhydroglucitol reflects glucose fluctuations. Therefore, it is plausible to postulate that glycemic variability may partially account for the significant association between 1,5-anhydroglucitol and ACPR. In accord, a recent study demonstrated that several measures of glycemic variability assessed by continuous glucose monitoring were significantly linked to glucagon-stimulated insulin secretion in Japanese patients with type 2 diabetes [32].

To our knowledge, we are the first to report this dose-response positive association between 1,5-anhydroglucitol and acute C peptide response. The major strength of our study is the accurate assessment of acute insulin response by AST. The relatively large sample size helps enhance the methodology and reliability of our findings. Apparently, several limitations should also be addressed. Firstly, because this is a cross-sectional study, the design does not allow us to look at the causal relationship between 1,5-anhydroglucitol and beta cell function. Thus, prospective study design is needed to assess this association. Secondly, our analyses adjusted for some confounding factors; however, unmeasured factors such as family history of diabetes, other related chronic diseases, dietary factors, and physical activity status could not be evaluated. Finally, only Chinese hospitalized patients with type 2 diabetes were enrolled in the study. Therefore, the generalizability of our findings to other populations is uncertain and needs to be tested in the future.

In conclusion, we demonstrated a dose-response linear association between 1,5-anhydroglucitol and ACPR. 1,5-Anhydroglucitol was likely to be associated with *β*-cell function. Further analysis with large sample size and prospective study design is warranted to validate our findings.

## Figures and Tables

**Table 1:** Characteristics of the study subjects.

Variables	1,5-Anhydroglucitol (*μ*g/mL)				*P* for trend
	<1.2	1.2-2.0	2.1-4.4	≥4.5	
No. of patients	150	159	156	158	
Age (years)	42.4 ± 12.2	47.9 ± 10.1	49.7 ± 10.1	52.1 ± 10.5	<0.001
Men, *n* (%)	110 (73.3)	126 (79.2)	109 (69.9)	92 (58.2)	0.001
Duration of diabetes (years)	6.03 ± 6.36	6.76 ± 6.08	8.47 ± 6.83	8.08 ± 6.01	0.001
BMI (kg/m^2^)	25.58 ± 3.72	25.19 ± 3.60	25.95 ± 3.55	25.72 ± 3.90	0.374
SBP (mmHg)	128 ± 16	129 ± 14	129 ± 16	129 ± 14	0.507
DBP (mmHg)	81 ± 10	82 ± 10	81 ± 9	79 ± 9	0.445
CRP (mg/dL)	2.00 ± 3.08	2.46 ± 3.90	2.14 ± 3.60	1.26 ± 1.77	0.023
TG (mmol/L)	2.21 ± 1.87	1.71 ± 1.24	1.89 ± 1.25	1.57 ± 0.78	<0.001
TC (mmol/L)	5.09 ± 1.14	4.87 ± 1.12	4.82 ± 1.01	4.56 ± 0.90	<0.001
LDL-c (mmol/L)	3.09 ± 1.06	3.07 ± 0.99	2.94 ± 0.83	2.76 ± 0.73	0.001
HDL-c (mmol/L)	1.00 ± 0.27	1.05 ± 0.26	1.06 ± 0.27	1.09 ± 0.29	0.005
eGFR (mL/min/1.73 m^2^)	85.0 ± 26.8	84.4 ± 25.9	92.4 ± 37.1	94.9 ± 32.6	0.001
FPG (mmol/L)	9.23 ± 2.56	8.71 ± 2.63	8.34 ± 2.66	6.50 ± 1.45	<0.001
2hPG (mmol/L)	14.89 ± 4.39	13.69 ± 4.38	13.03 ± 4.08	10.36 ± 3.35	<0.001
HbA1c (%)	11.5 ± 1.9	10.5 ± 1.8	9.5 ± 1.7	7.3 ± 1.2	<0.001
GA (%)	31.8 ± 7.40	28.9 ± 7.11	24.0 ± 6.56	18.3 ± 4.48	<0.001
ARCP (ng/mL)	1.26 ± 0.82	1.31 ± 0.78	1.41 ± 0.85	1.58 ± 0.93	<0.001
Glucose-lowering drugs, *n* (%)					
Insulin	150 (100.0)	157 (98.7)	141 (90.4)	104 (65.8)	<0.001
Metformin	49 (32.7)	57 (35.8)	59 (37.8)	79 (50.0)	0.001
TZDs	0 (0.00)	0 (0.00)	4 (2.6)	9 (5.7)	0.001
*α*-Glucosidase inhibitors	18 (12.0)	20 (12.6)	32 (20.5)	35 (22.2)	0.002

**Table 2:** Odds ratios for impaired *β*-cell function assessed by arginine stimulation test across different levels of 1,5-anhydroglucitol by logistic regression analysis.

	1,5-Anhydroglucitol (*μ*g/mL)				*P* for trend	As a continuous variable^∗^
	<1.2	1.2-2.0	2.1-4.4	≥4.5		
No. of patients	150	159	156	158		
No. of cases	131	136	126	123		
Age-adjusted	1.00	0.66 (0.34-1.25)	0.50 (0.27-0.93)	0.35 (0.19-0.65)	0.013	0.47 (0.29-0.77)
Multivariable-adjusted^#^	1.00	0.47 (0.23-0.99)	0.41 (0.20-0.84)	0.27 (0.13-0.57)	0.042	0.40 (0.23-0.71)

^∗^Transformed by logarithm.

^#^Multivariable adjustments included age, sex, diabetes duration, BMI, SBP, TG, LDL-c, HDL-c, eGFR, and glucose-lowering therapies except insulin.

**Table 3:** Linear regression analysis for ACPR by different confounding factors.

Independent variables	*β* (95% confidence interval)	SE	Standardized *β*	*P*
Duration of diabetes	-0.024 (-0.034 to -0.013)	0.005	-0.177	<0.001
BMI	0.072 (0.054 to 0.089)	0.009	0.307	<0.001
1,5-Anhydroglucitol	0.421 (0.278 to 0.563)	0.073	0.211	<0.001

Covariates included age, sex, diabetes duration, BMI, SBP, TG, LDL-c, HDL-c, eGFR, and glucose-lowering therapies except insulin.

## Data Availability

The datasets generated during and/or analyzed during the current study are not publicly available but are available from the corresponding author on reasonable request.

## Supplementary Materials

Supplementary Materials The Supplementary figure includes the flow chart of the study.
