# Supplementary figures and images for: Association between 1,5-Anhydroglucitol and Acute C Peptide Response to Arginine among Patients with Type 2 Diabetes
Source: J Diabetes Res. 2020 Jul 21;2020:4243053. doi: 10.1155/2020/4243053 (PMC7391082; doi:10.1155/2020/4243053)

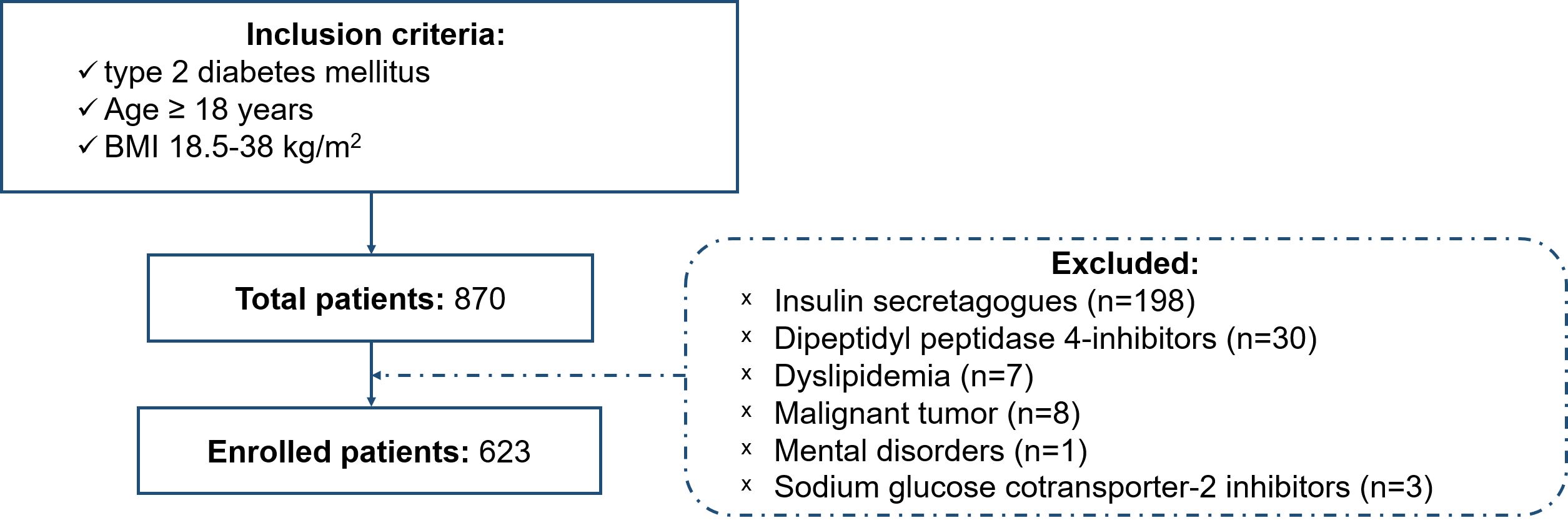

Supplement: Supplementary Materials — The Supplementary figure includes the flow chart of the study. [file 4243053.f1.tif]
